# Successful Application of Argatroban During VV-ECMO in a Pregnant Patient Complicated With ARDS due to Severe Tuberculosis: A Case Report and Literature Review

**DOI:** 10.3389/fphar.2022.866027

**Published:** 2022-07-11

**Authors:** Hongxia Wu, Yongjiang Tang, Xiaofeng Xiong, Min Zhu, He Yu, Deyun Cheng

**Affiliations:** Department of Respiratory and Critical Care Medicine, West China Hospital, Sichuan University, Chengdu, China

**Keywords:** extracorporeal membrane oxygenation, pregnancy, tuberculosis, thrombocytopenia, argatroban, anticoagulation

## Abstract

Severe tuberculosis during pregnancy may progress to acute respiratory distress syndrome (ARDS), and venovenous (VV) extracorporeal membrane oxygenation (ECMO) should be considered if conventional lung-protective mechanical ventilation fails. However, thrombocytopenia often occurs with ECMO, and there are limited reports of alternative anticoagulant therapies for pregnant patients with thrombocytopenia during ECMO. This report describes the first case of a pregnant patient who received argatroban during ECMO and recovered. Furthermore, we summarized the existing literature on VV-ECMO and argatroban in pregnant patients. A 31-year-old woman at 17 weeks of gestation was transferred to our hospital with ARDS secondary to severe tuberculosis. We initiated VV-ECMO after implementing a protective ventilation strategy and other conventional therapies. Initially, we selected unfractionated heparin anticoagulant therapy. However, on ECMO day 3, the patient’s platelet count and antithrombin III (AT-III) level declined to 27 × 10^3^ cells/μL and 26.9%, respectively. Thus, we started the patient on a 0.06 μg/kg/min argatroban infusion. The argatroban infusion maintenance dose ranged between 0.9 and 1.2 μg/kg/min. The actual activated partial thromboplastin clotting time and activated clotting time ranged from 43 to 58 s and 220–260 s, respectively, without clinically significant bleeding and thrombosis. On day 27, the patient was weaned off VV-ECMO and eventually discharged. VV-ECMO may benefit pregnant women with refractory ARDS, and argatroban may be an alternative anticoagulant for pregnant patients with thrombocytopenia and AT-III deficiency during ECMO.

## Introduction

According to the World Health Organization, 32% of women among all age groups are affected by tuberculosis (TB) (World Health Organization, 2020). In addition, acute respiratory distress syndrome (ARDS), which is commonly observed in patients with miliary tuberculosis (Agarwal et al., 2005; Kim et al., 2008), makes women more susceptible if they are pregnant (Lapinksy, 2015; Moore et al., 2016). Thus, poor prognoses for the mother and fetus have been associated with maternal TB. Consequently, ARDS is a leading cause of non-obstetric mortality during pregnancy.

Extracorporeal membrane oxygenation (ECMO) use has increased dramatically in recent years. Venous-arterial ECMO provides cardiopulmonary support to patients, whereas venovenous ECMO (VV-ECMO) provides only respiratory support, improving oxygenation and hypercapnia when conventional therapies fail (Acute Respiratory Distress Syndrome Network et al., 2000; Guérin et al., 2013; Spieth and Zhang, 2016). In addition, VV-ECMO is a powerful tool for avoiding oxygen- and ventilator-induced lung injury by resting the lungs (Vaquer et al., 2017; Pacheco et al., 2018). Therefore, VV-ECMO should be considered for refractory ARDS (Clarence et al., 2012).

Although ECMO therapy usually requires anticoagulation therapy, and unfractionated heparin (UFH) is the most used anticoagulant (Terragni et al., 2014), argatroban can be used as an alternative anticoagulant in patients with UFH contraindications, such as heparin-induced thrombocytopenia (HIT) (Coughlin and Bartlett, 2015).

Nonetheless, using VV-ECMO for pregnant patients with ARDS is limited, especially in cases complicated by thrombocytopenia. Even though some studies have reported using argatroban to treat deep vein thrombosis (DVT) and acute pulmonary embolism (PE) in a pregnant population with HIT, no study has directly reported using argatroban for pregnant patients with thrombocytopenia during ECMO. Therefore, we report the first case of a pregnant patient with ARDS secondary to severe TB and thrombocytopenia requiring ECMO support who received argatroban as a substitute for UFH. Notably, the patient was successfully weaned off ECMO without severe bleeding or thrombosis, recovered, and was discharged from the hospital.

## Case Presentations

The present study was confirmed by the Committee on Medical Ethics of West China Hospital, Sichuan University. The study was accomplished according to the Declaration of Helsinki and CARE guidelines with the written informed consent of the patient’s guardian.

A 31-year-old woman at 17 weeks of gestation was admitted to the emergency department for cough, expectoration for 1 month, and dyspnea and fever for 3 days. A previous hospital administered empirical anti-infection therapy with ceftriaxone, but the effects were unsatisfactory. Furthermore, two days later, non-invasive mechanical ventilation failed due to ARDS. Consequently, endotracheal intubation and invasive mechanical ventilation were performed, and the patient was admitted to the medical intensive care unit (MICU). The patient had a history of chronic hepatitis B, long-term oral antiviral treatment, three pregnancies, and two induced abortions.

Table 1 summarizes the vital signs and general laboratory test results. At admission, the vital signs were: temperature, 39.5°C; blood pressure, 97/43 mm Hg (continuous intravenous pumping of norepinephrine 1.0 μg/kg/min); respiratory rate, 40°bpm; heart rate, 145°bpm; hemoglobin oxygen saturation, 80%; and a fraction of inspired oxygen (FiO_2_), 100%. The patient was 159 cm tall and weighed 52.5 kg. Physical examination revealed shortness of breath, wet voice, wheezing of both lungs, and tachycardia. Gastrointestinal decompression drained a small amount of dark red blood cells.

**TABLE 1:** Findings from vital signs and laboratory tests at admission and discharge.

Findings	Parameters	At admission	At discharge
Vital signs	Temperature (°C)	39.5	36.5
	Heart rate (times/min)	145	105
	Respiratory rate (times/min)	40	22
	Blood pressure (mmHg)	97/43	120/60
Arterial blood gas analysis	PH	7.399	7.431
	PO_2_ (mmHg)	58.4	98
	PCO_2_ (mmHg)	41.9	38.4
	PO_2_/FiO_2_	58.4	330
	Lac (mmol/L)	2.1	1.6
General laboratory tests	WBC (cells/μL)	5,340	6,410
	PLT (cells/μL)	13,600	403,000
	N %	90.8	65.6
	HGB (g/L)	102	112
	PCT (ng/L)	1.4	0.05
	CRP (mg/L)	94	5.37
	Albumin (g/L)	25.3	41.5
	TBIL (μmol/L)	8.8	12.7
	DBIL (μmol/L)	3.1	9.2
	AST (U/L)	35	30
	ALT (U/L)	72	34
	BUN (mmol/L)	14.3	7.4
	CR (μmol/L)	39	23
	eGFR (ml/min/1.73m^2^)	134.82	156.06
	Potassium (mmol/L)	3.05	4.1
	Sodium (mmol/L)	125.6	136
	Chlorine (mmol/L)	98.4	97.3
	BNP (ng/L)	1871	346
	PT (s)	10.3	11.2
	APTT (s)	36.5	26.8
	INR	1.61	0.98
	AT-III (%)	76.7	96.3
	FIB (g/L)	4.63	2.84
	D-dimer (mg/L)	2.6	1.35
	CD4^+^ T cell (cells/μL)	63	246

PH, pondus hydrogenii; PO_2_, partial pressure of oxygen; PCO_2_, partial pressure of carbon dioxide; PO_2_/FiO_2_, partial pressure of oxygen/fraction of inspiration oxygen; Lac, lactic acid. WBC, white blood cells; N %, percentage of neutrophil; PLT, platelet; HGB, hemoglobin; PCT, procalcitonin; CRP, C-reactive protein; AST, aspartate aminotransferase; ALT, alanine aminotransferase; BUN, blood urea nitrogen; CR, creatinine; eGFR, estimated glomerular filtration rate; PT, prothrombin time; APTT, activated partial thromboplastin time; INR, international normalized ratio; AT-III, antithrombin III; FIB, fibrinogen.

We also performed laboratory tests; smears and cultures for acid-fast bacilli, TB-DNA, and TB-Xpert of bronchoalveolar lavage fluid (BALF) tests were positive. In addition, TB interferon-gamma release assay, purified protein derivative skin test, and hepatitis B surface antigen and core antibody results were positive. Next-generation sequencing (NGS) of BALF and peripheral blood revealed 235 and 75 sequences of *Mycobacterium tuberculosis* complex, respectively. However, blood cultures for aerobic and anaerobic bacteria were negative, as tested for G and GM, human immunodeficiency virus, hepatitis C, Epstein-Barr virus, cytomegalovirus, *Mycoplasma pneumoniae*, and TORCH (i.e., toxoplasmosis, other, rubella, cytomegalovirus, and herpes) toxoplasma. Also, the HBV DNA levels were below the lower limit of detection.

**FIGURE 1:**
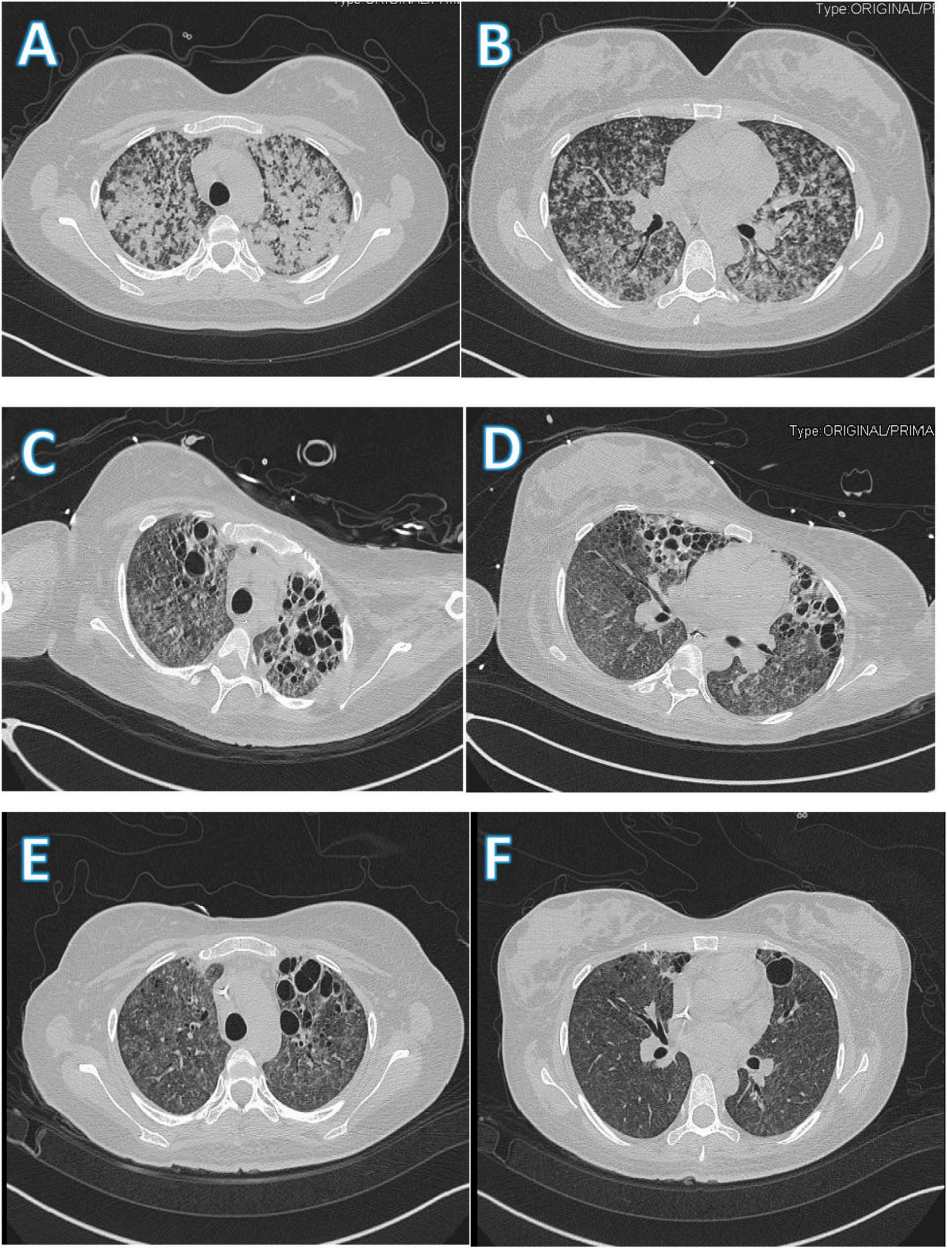
Image of chest CT scan. **(A,B)** chest CT on admission showed multiple miliary nodular lesions that were partially clustered and consolidated diffusely in both lungs; **(C,D)** After 36 days of treatment, chest CT showed absorption of miliary nodular lesions and new vesicles in the bilateral lung; **(E,F)** After 56 days of treatment, chest CT showed absorption of miliary nodular lesions and vesicles in the bilateral upper lung.

Nevertheless, high-resolution computed tomography (CT) showed multiple miliary nodular lesions partially clustered and diffusely consolidated in both lungs (Figures 1A, B). Moreover, obstetric ultrasonography revealed a normal fetal heart rate. Finally, head CT, echocardiography, and limb vascular ultrasound findings were normal.

The initial VV-ECMO treatment, which began after conventional therapies for ARDS, failed. The initial ECMO parameters were: blood flow, 5.5 L/min; centrifugal pump speed, 4,010 circles/min; water temperature, 37°C; the fraction of delivered oxygen, 100%; oxygen flow 3.5 L/min; invasive mechanical ventilation, A/C (VC); respiratory rate, 12 times/min; tidal volume, 280 ml; positive end-expiratory pressure (PEEP), 5 cmH_2_O; and FiO_2_, 60%. UFH was initially selected for anticoagulant therapy; 12,500 units of UFH were administered via bolus at the cannulation time, followed by a continuous infusion of 10 units/kg/h. The active partial thromboplastin time (APTT) and activated clotting time (ACT) therapeutic targets were 40–60 s and 200–240 s, respectively. APTT and ACT were initially checked every 2 h, followed by every 4 h after reaching the therapeutic target. If these two indicators were inconsistent, the APTT value was preferentially considered.

After 3 days of VV-ECMO, the patient’s platelet count and antithrombin III (AT-III) level decreased to 27 × 10^3^ cells/μL and 26.9%, respectively, and the 4T (the pretest probability of heparin-induced thrombocytopenia) score was 5. However, the platelet antibody test results were negative. Therefore, we initiated argatroban as an alternative therapy on ECMO day 3, administering argatroban by an infusion of 0.06 μg/kg/min without a bolus. The APTT and ACT therapeutic targets and checks were identical to those for the UFH therapy.

**FIGURE 2:**
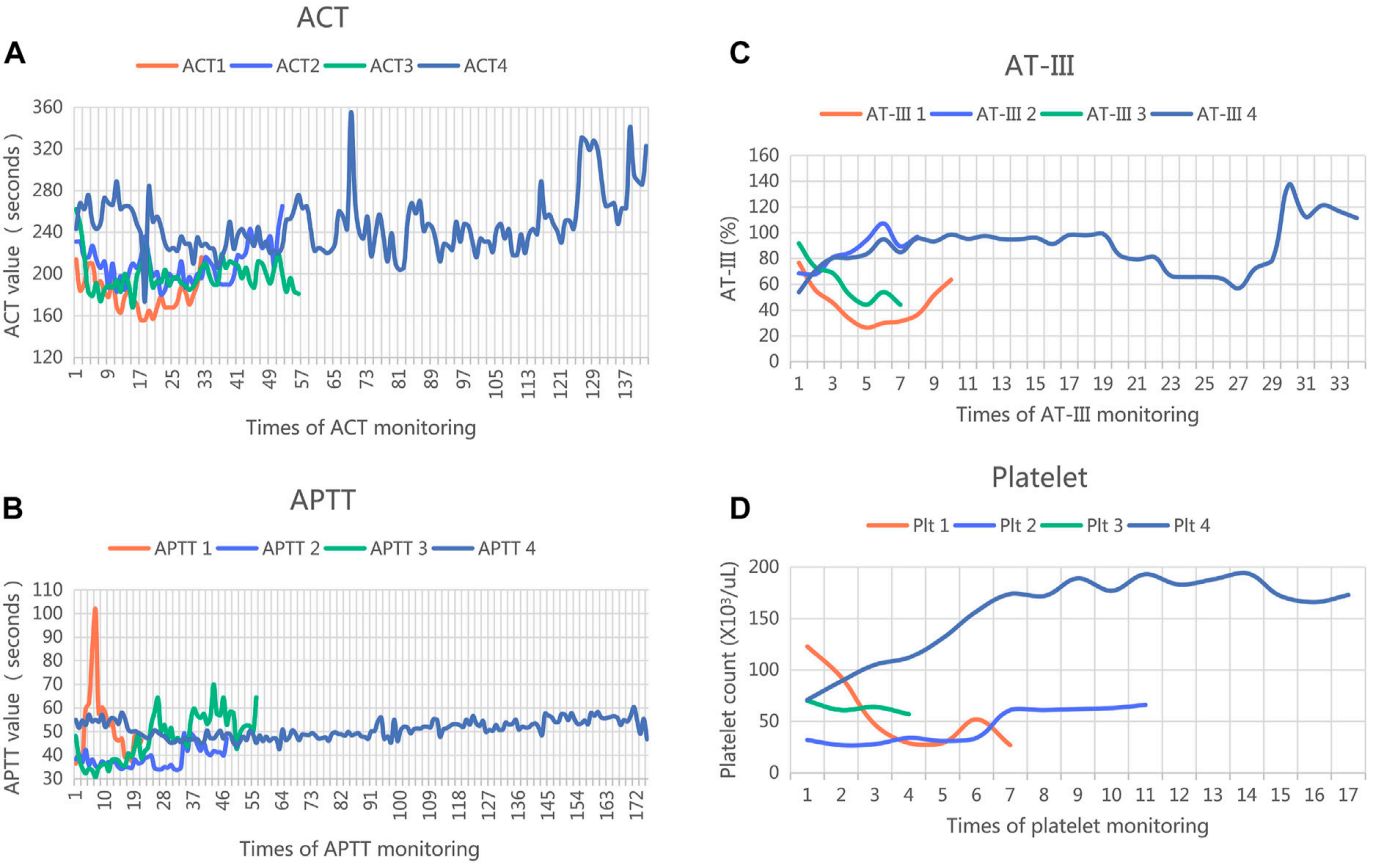
Monitoring of ACT, APTT, AT-III and platelet in anticoagulant therapy. ACT, activated clotting time; UFH, unfractionated heparin; APTT, active partial thromboplastin time; AT-III, anti-thrombin III; **(A)** ACT 1 (orange line) showed ACT level during day 1 to day 3 (UHF therapy); ACT 2 (blue line) showed ACT level during day 3 to day 8 ( argatroban therapy); ACT 3 (green line) showed ACT level during day 8 to day 12 ( UHF therapy); ACT 4 (dark blue line) showed ACT level during day 12 to day 27 (argatroban therapy); **(B)** APTT 1 (orange line) showed APTT level during day 1 to day 3 (UHF therapy); APTT 2 (blue line) showed APTT level during day 3 to day 8 (argatroban therapy); APTT 3 (green line) showed APTT level during day 8 to day 12 ( UHF therapy); APTT 4 (dark blue line) showed APTT level during day 12 to day 27 (argatroban therapy); **(C)** AT-III 1 (orange line) showed AT-III level during day 1 to day 3 (UHF therapy); AT-III 2 (blue line) showed AT-III level during day 3 to day 8 (argatroban therapy); AT-III 3 (green line) showed AT-III level during day 8 to day 12 ( UHF therapy); AT-III 4 (dark blue line) showed AT-III level during day 12 to day 27 (argatroban therapy); **(D)** platelet 1 (orange line) showed platelet level during day 1 to day 3 (UHF therapy); platelet 2 (blue line) showed platelet level during day 3 to day 8 (argatroban therapy); platelet 3 (green line) showed platelet level during day 8 to day 12 ( UHF therapy); platelet 4 (dark blue line) showed platelet level during day 12 to day 27 (argatroban therapy).

After platelet infusion and alternative anticoagulant therapy, the patient’s platelet count increased to 66 × 10^3^ cells/μL on day 8. Thus, UFH was resumed. However, we reverted to argatroban on day 11 due to a decreased platelet count and unsatisfactory APTT with UFH. The APTT and ACT ranged from 43 to 58 s and 220–260 s, respectively, and the argatroban infusion varied from 0.9 to 1.2 μg/kg/min. However, the platelet count and AT-III level were within the normal range in the subsequent therapy. In addition, hematuria appeared during argatroban therapy but improved without additional treatment. Notably, the patient met the therapeutic targets during argatroban therapy without circuit thrombosis, severe bleeding, infection, hemolysis, or thrombocytopenia. In addition, continuous ultrasound monitoring did not find fetal distress. Figure 2 presents the APTT, ACT, AT-III, and platelet count variations throughout the anticoagulant treatments.

Moreover, we prescribed the isoniazid, rifampicin, pyrazinamide, ethambutol (HRZE) regimen and dexamethasone as anti-TB therapies. However, on day 7 of the HRZE regimen, the patient developed a systemic skin rash and liver damage (alanine aminotransferase 143 U/L, total bilirubin 88 μmol/L). Consequently, we changed to the ethambutol, levofloxacin, amikacin, cycloserine, and linezolid regimen based on liver protection and anti-allergy treatments and decreased then ceased dexamethasone for 1 month. We also prescribed meropenem empiric antibacterial treatment and sedation, analgesia, muscle relaxation therapy, functional vascular medicine, liver protection therapy, proton-pump inhibitors, and enteral nutrition after MICU admission.

Under the above treatments, the patient’s temperature and oxygenation gradually improved. However, on day 23, the patient developed a fever. In addition, blood and BALF NGS revealed *Klebsiella pneumoniae*, and a drug-sensitivity test determined that the organism was multidrug-resistant. Therefore, 2.5 g q8h of Zavicefta was prescribed based on the drug susceptibility results. However, after eight days of initiating Zavicefta treatment, the patient’s temperature did not decrease; hence, we had to administer tigecycline. Later, the patient’s temperature gradually returned to normal after the combined drug therapy.

Adequate oxygenation and ventilation were provided to the patient during the downregulation of ECMO parameters, and on day 27, the patient was weaned off VV-ECMO successfully along with administration of low-molecular-weight heparin, followed by argatroban for hypercoagulability. Finally, on day 32, we performed a tracheotomy. However, on day 38, after oxygenation, we weaned the patient off mechanical ventilation and observed an improvement in the chest images (Figures 1C,D).

After anti-TB and other treatments, the patient and family members decided to terminate the pregnancy. However, on day 42, the patient underwent labor induction, delivering a dead fetus, and on day 45, the patient experienced a sudden twitch and disturbance of consciousness, which continued for 1 minute. However, emergency head CT showed no signs of hemorrhage or infarction. Additional tests conducted included a lumbar puncture for cerebrospinal fluid (CSF; 123 mmH_2_O of pressure), routine CSF analyses (identifying no nucleated cells or erythrocytes), and CSF biochemical analysis (protein 0.66 g/L, glucose 3.88 mmol/L, and chlorine 120 mmol/L). Besides, synchronous blood glucose and chlorine were 5.36 mmol/L and 117 mmol/L, respectively, and the immunoglobulin G synthesis rate was 4.205 mg/day. At the same time, smears and cultures of acid-fast bacilli and other bacteria and ink stains, cryptococcal antigens, TB-DNA, and TB-Xpert tests were negative. However, head magnetic resonance imaging indicated multiple demyelinating brain white matter lesions. Therefore, we prescribed levetiracetam (0.5 g) twice daily, and consequently, the patient did not experience more twitches.

On day 49, the patient was transferred to the TB ward without respiratory support or active vascular drugs. The anti-TB treatment regimen was changed to the ethambutol, rifapentine, amikacin, and linezolid regimen to account for potential nervous system adverse reactions to cycloserine and levofloxacin. The patient’s symptoms and chest images improved significantly (Figures 1E,F), and she was discharged on day 59.

## Discussion

The article reported the case of a 31-year-old pregnant woman admitted to our institution with ARDS secondary to severe TB. VV-ECMO was initiated following conventional strategies, with argatroban being administered during ECMO when the patient had thrombocytopenia and AT-III deficiency. However, the patient was successfully weaned off ECMO and recovered well.

ECMO use has increased dramatically in recent years, although ECMO during pregnancy has been associated with an increased risk of maternal and fetal bleeding complications. However, some studies have reported that ECMO is safe and effective in select pregnant patients. For example, a review of 41 pregnant patients with ARDS due to severe H1N1-influenza receiving ECMO reported that the maternal and fetal survival rates were 77.8 and 65%, respectively (Moore et al., 2016). The study further concluded that ECMO safety during pregnancy was comparable to non-pregnant patients and thereby safe for the mother and fetus. Another study reported four pregnant patients with respiratory or cardiorespiratory failure requiring ECMO therapy (Sharma et al., 2015), which used heparin as the anticoagulant in the initial stages. It also maintained ACT, APTT, and thromboelastography between 160 and 180 s, 50 and 80 s, and 2.5 to 3.0 times the standard reaction time. However, none of the patients developed severe hemorrhage. The authors also reviewed 67 patients during pregnancy, and postpartum supported by extracorporeal life support and found that the maternal and fetal survival rates were 80 and 70%, respectively.

Moreover, an observational study found that ECMO benefited obstetric patients with ARDS due to H1N1-influenza (Australia and New Zealand Extracorporeal Membrane Oxygenation Influenza Investigators et al., 2009). In addition, a meta-analysis reported that the survival rate of obstetrical patients who received ECMO for ARDS due to H1N1-influenza was 75% (Saad et al., 2016). Hence, pregnancy is not an absolute contraindication to ECMO. Another study summarized the potential indications for VV-ECMO during pregnancy and the postpartum period, reporting that 1) severe but reversible respiratory failure, 2) hypercapnia with severe respiratory acidosis, and 3) partial pressure of oxygen to FiO_2_ ratio of less than 100 with a FiO_2_ value of ≥0.9 and a PEEP value of ≥10 cmH_2_O was suitable indications (Pacheco et al., 2018). However, no convincing evidence has shown that VV-ECMO can improve outcomes in obstetrical patients with severe ARDS, indicating the requirement for further trials.

UFH is the most used anticoagulant but HIT and acquired AT-III deficiency are contraindications to UFH therapy. However, direct thrombin inhibitors, such as argatroban and bivalirudin (Young et al., 2004; Schaden and Kozek-Langenecker, 2010; Scott and Webster, 2014; Sanfilippo et al., 2017; Koster et al., 2018), are UFH alternatives (Coughlin and Bartlett, 2015; Burstein et al., 2019). For example, a systematic review identified 307 participants treated with argatroban during ECMO (Geli et al., 2021). In most studies, argatroban was continuously infused without a loading dose. Furthermore, the initial dose varied from 0.05 to 2 μg/kg/min and was titrated to achieve the target therapeutic range. Most studies chose APTT as a monitoring index, but some used ACT. The optimal APTT therapeutic targets were 43–70 s and 60–100 s, and the optimal ACT therapeutic targets were 150–210 s and 180–230 s (Geli et al., 2021). Furthermore, the bleeding and thrombosis complication rates for argatroban were comparable to those of UFH. However, evidence from the obstetrical population is limited.

Argatroban is a pregnancy category index B drug, and the FDA recommends using these drugs carefully and only when there is a clear indication. Argatroban has been deemed safe in animal reproduction control studies, but its safety in pregnant women remains unclear. Furthermore, no study has directly reported argatroban use during ECMO in pregnant patients. Some, however, have reported on argatroban use in patients with DVT and acute PE, and one study reported using argatroban for a pregnant patient undergoing pulmonary embolectomy with cardiopulmonary bypass (Taniguchi et al., 2008). In that report, argatroban was administered at a bolus dose of 0.1 mg/kg, then continuously infused at 5-10 μg/kg/min, and ACT was maintained over 350 s (Furukawa et al., 2001). Resultantly, the patient recovered and delivered a baby.

Another case report described a pregnant woman with DVT and HIT (Ekbatani et al., 2010). Argatroban infusion was initiated at 2 μg/kg/min, and the maintenance dose was titrated (max 10 μg/kg/min) to 1.5 times the normal range of APTT. The argatroban infusion was discontinued before neuraxial analgesia, and the patient underwent routine labor and delivery. Another study also described a 33-year-old woman with hereditary AT deficiency type I who developed DVT at 7 weeks of gestation (Tanimura et al., 2012). HIT appeared during the UFH therapy; thus, argatroban and AT infusion were prescribed. The argatroban infusion rate ranged from 2.5 to 4.5 μg/kg/min to achieve an APTT with 1.5–2.0 times control. In the end, the patient delivered without adverse effects of argatroban. Finally, we identified one case of portal vein thrombosis and possible HIT treated with argatroban during pregnancy (Young et al., 2008). Argatroban infusion was initiated at 2 μg/kg/min, and the dosage was titrated (2–8 μg/kg/min) based on the APTT. The treatment continued until labor was induced, and the patient delivered the baby without complications and was without adverse effects of argatroban.

Nonetheless, no reports have directly discussed argatroban use during ECMO in obstetrical populations with ARDS; our report is the first. In our case, the patient was successfully weaned from ECMO and recovered from severe TB complicated by ARDS without serious complications from anticoagulation therapy.

In our report, the APTT values were more stable than the ACT values, and the stability and APTT target during the argatroban period seemed better than those during the UHF period. Furthermore, anticoagulation therapy with argatroban did not influence the platelet count or AT-III. However, there is no consensus on the optimal anticoagulation strategy for pregnant patients, with varied observations on argatroban usage, dose, monitoring index, and target values among previous studies. There was, however, one commonality in our report and prior studies on the pregnant population; the maintenance dose of argatroban for pregnant patients was greater than that for the general population. ECMO is a complex therapy affecting medications' pharmacokinetics (PK) and pharmacodynamics (PD). Thus, optimal pharmacological management of ECMO remains difficult. In addition, the altered physiology of pregnancy and critical illness status may also affect the PK and PD of medications. Close observation of bleeding, coagulation index detection, and drug efficacy evaluation are the key points in the care of VV-ECMO during pregnancy. Therefore, these issues require further confirmation in clinical trials. Furthermore, there is inconclusive data regarding the efficacy and safety of argatroban applied during VV-ECMO in the obstetrical population, and the optimal dose and targeted APTT and ACT ranges remain unclear, requiring further research.

## Conclusion

To summarize, VV-ECMO may be considered for pregnant patients with ARDS refractory to conventional treatment. In addition, argatroban may be an alternative anticoagulant for pregnant patients with thrombocytopenia and AT-III deficiency during ECMO. However, more data is needed to clarify the specific parameters.

## Data Availability

The original contributions presented in the study are included in the article/Supplementary Material. Further inquiries can be directed to the corresponding authors.

## Abbreviations

ARDS, acute respiratory distress syndrome; ECMO, extracorporeal membrane oxygenation; VV-ECMO, venovenous ECMO; VA-ECMO, venous-arteria ECMO; HIT, heparin-induced thrombocytopenia; UFH, unfractionated heparin; DVT, deep vein thrombosis; PE, pulmonary embolism; BALF, bronchoalveolar lavage fluid; NGS, next-generation sequencing.
