# Supplementary material for: Successful Application of Argatroban During VV-ECMO in a Pregnant Patient Complicated With ARDS due to Severe Tuberculosis: A Case Report and Literature Review
Source: Front Pharmacol. 2022 Jul 11;13:866027. doi: 10.3389/fphar.2022.866027 (PMC9309810; doi:10.3389/fphar.2022.866027)
Supplement: Supplementary file 1 [file Table3.DOCX]

| Findings | Parameters | At admission | At discharge |
| --- | --- | --- | --- |
| Vital signs | Temperature (℃) | 39.5 | 36.5 |
|  | heart rate (times/min) | 145 | 105 |
|  | respiratory rate (times/min) | 40 | 22 |
|  | blood pressure (mmHg) | 97/43 | 120/60 |
| Arterial blood gas analysis | PH | 7.399 | 7.431 |
|  | PO2 (mmHg) | 58.4 | 98 |
|  | PCO2 (mmHg) | 41.9 | 38.4 |
|  | PO2/FiO2 | 58.4 | 330 |
|  | Lac (mmol/L) | 2.1 | 1.6 |
| General laboratory tests | WBC (cells /uL) | 5,340 | 6,410 |
|  | PLT (cells /uL) | 13,600 | 403,000 |
|  | N % | 90.8 | 65.6 |
|  | HGB (g/L) | 102 | 112 |
|  | PCT (ng/L) | 1.4 | 0.05 |
|  | CRP (mg/L) | 94 | 5.37 |
|  | Albumin (g/L) | 25.3 | 41.5 |
|  | TBIL (umol/L) | 8.8 | 12.7 |
|  | DBIL (umol/L) | 3.1 | 9.2 |
|  | AST (U/L） | 35 | 30 |
|  | ALT (U/L） | 72 | 34 |
|  | BUN (mmol/L) | 14.3 | 7.4 |
|  | CR (umol/L) | 39 | 23 |
|  | eGFR (ml/min/1.73m^2^) | 134.82 | 156.06 |
|  | Potassium (mmol/L) | 3.05 | 4.1 |
|  | Sodium (mmol/L) | 125.6 | 136 |
|  | Chlorine (mmol/L) | 98.4 | 97.3 |
|  | BNP (ng/L) | 1871 | 346 |
|  | PT (s) | 10.3 | 11.2 |
|  | APTT (s) | 36.5 | 26.8 |
|  | INR | 1.61 | 0.98 |
|  | AT-III (%) | 76.7 | 96.3 |
|  | FIB (g/L) | 4.63 | 2.84 |
|  | D-dimer (mg/l) | 2.6 | 1.35 |
|  | CD4+ T cell (cells/uL) | 63 | 246 |

Table 1 Findings from vital signs and laboratory tests at admission and discharge.

PH, pondus hydrogenii; PO2, partial pressure of oxygen; PCO2, partial pressure of carbon dioxide; PO2/FiO2, partial pressure of oxygen/fraction of inspire oxygen; Lac, lactic acid. WBC: white blood cells; N %, percentage of neutrophil; PLT: platelet; HGB: hemoglobin;PCT, procalcitonin; CRP: C-reactive protein; AST, aspartate aminotransferase; ALT, alanine aminotransferase; BUN, blood urea nitrogen; CR, creatinine; eGFR, estimated glomerular filtration rate; PT, prothrombin time; APTT, activated partial thromboplastin time; INR, International Normalized Ratio; AT-III, Antithrombin III; FIB, fibrinogen.
