# Supplementary material for: Successful Application of Argatroban During VV-ECMO in a Pregnant Patient Complicated With ARDS due to Severe Tuberculosis: A Case Report and Literature Review
Source: Front Pharmacol. 2022 Jul 11;13:866027. doi: 10.3389/fphar.2022.866027 (PMC9309810; doi:10.3389/fphar.2022.866027)
Supplement: Supplementary file 2 [file Table4.DOCX]

**A**

**B**


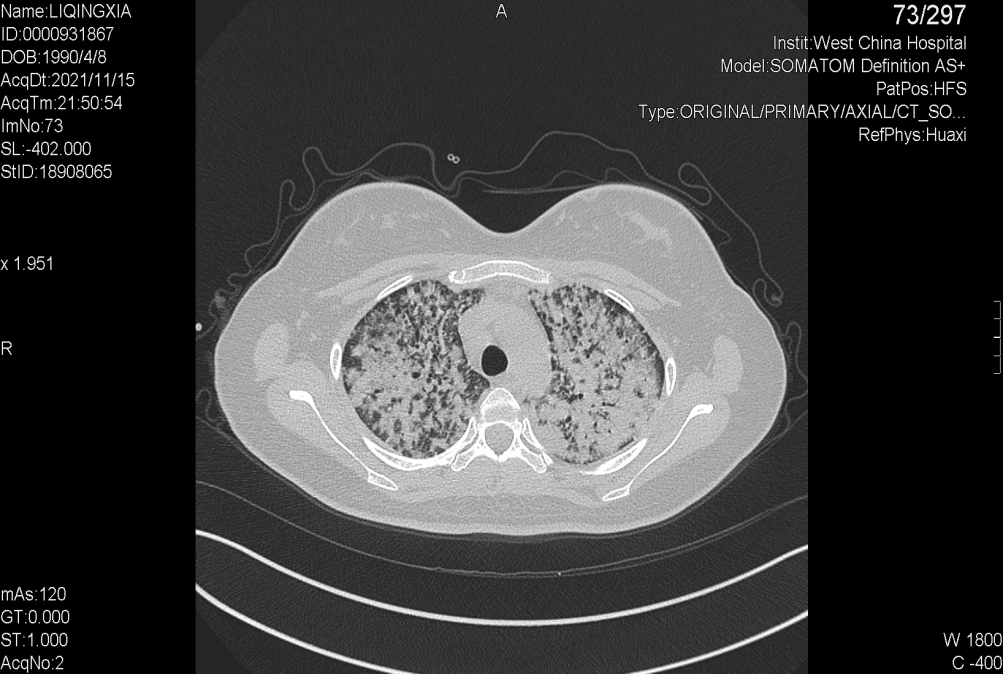

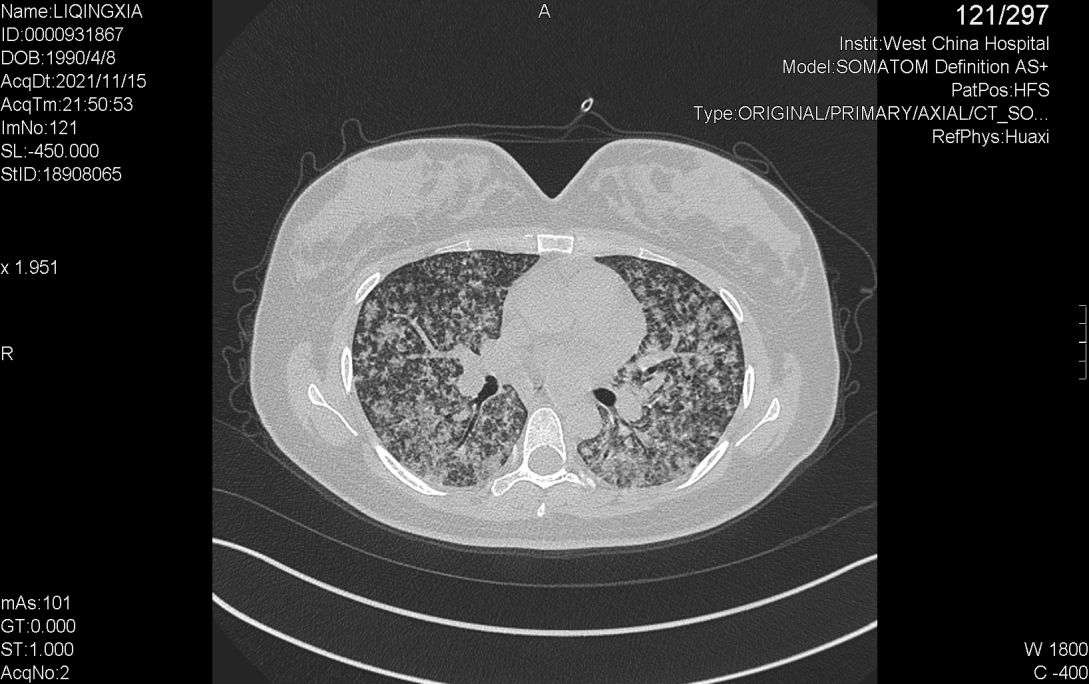


**D**

**C**


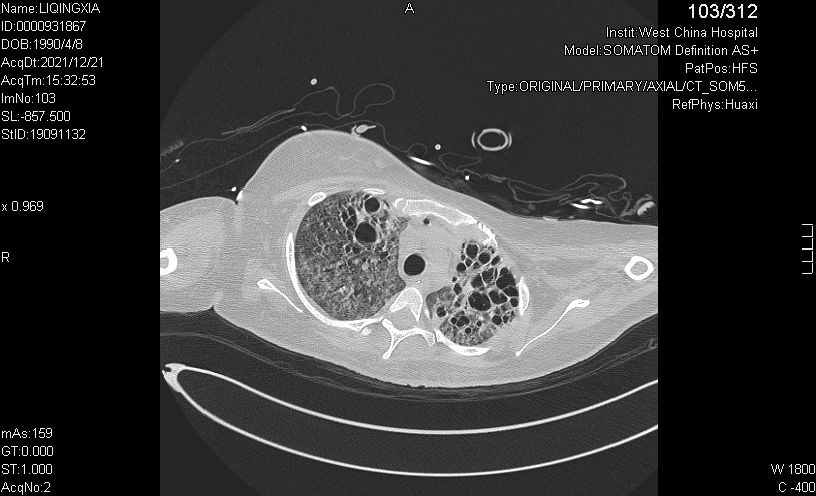

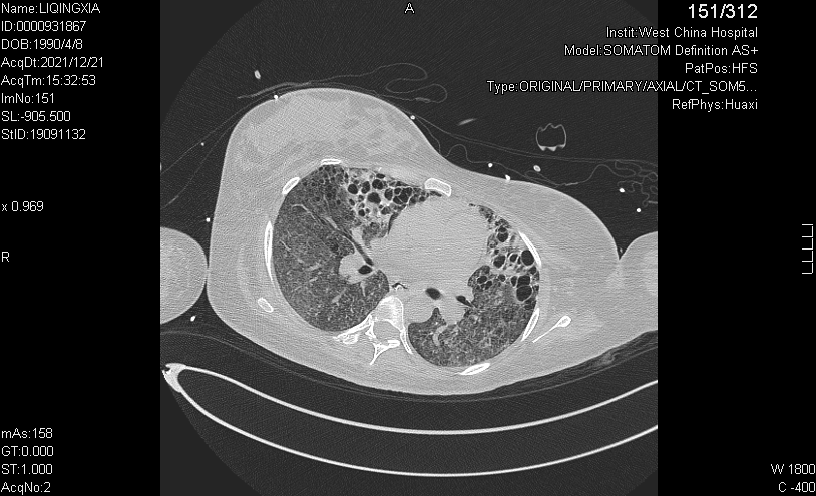


**E**

**F**


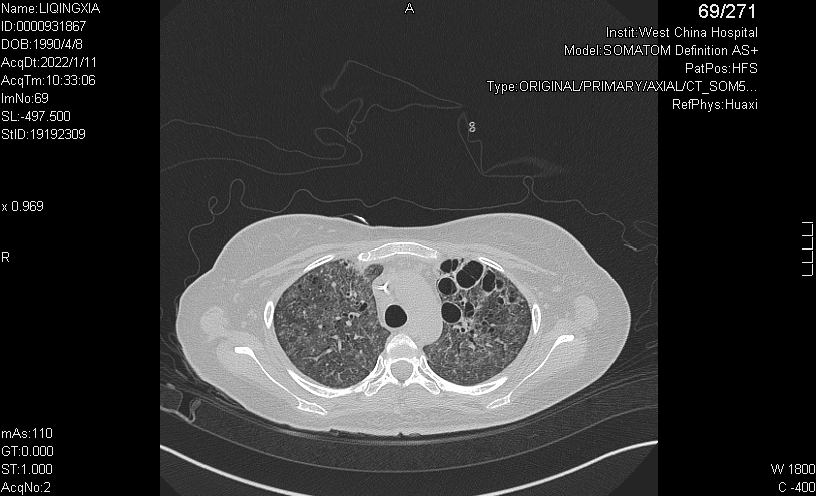

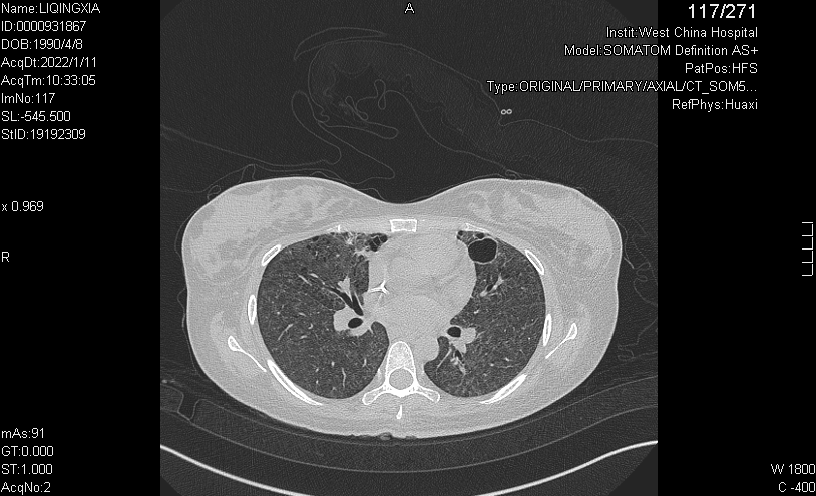


Figure 1. Image of chest CT scan. (A and B) chest CT on admission showed multiple miliary nodular lesions that were partially clustered and consolidated diffusely in both lungs; (C and D) After 36 days of treatment, chest CT showed absorption of miliary nodular lesions and new vesicles in the bilateral lung; (E and F) After 56 days of treatment, chest CT showed absorption of miliary nodular lesions and vesicles in the bilateral upper lung.

**A**

**B**

**C**

**D**

Figure 2. Monitoring of ACT, APTT, AT-III and platelet in anticoagulant therapy.

ACT, activated clotting time; UFH, unfractionated heparin; APTT, active partial thromboplastin time; AT-III, anti-thrombin III; (A) ACT 1 (orange line) showed ACT level during day 1 to day 3 (UHF therapy); ACT 2 (blue line) showed ACT level during day 3 to day 8 ( argatroban therapy); ACT 3 (green line) showed ACT level during day 8 to day 12 ( UHF therapy); ACT 4 (dark blue line) showed ACT level during day 12 to day 27 (argatroban therapy); (B) APTT 1 (orange line) showed APTT level during day 1 to day 3 (UHF therapy); APTT 2 (blue line) showed APTT level during day 3 to day 8 ( argatroban therapy); APTT 3 (green line) showed APTT level during day 8 to day 12 ( UHF therapy); APTT 4 (dark blue line) showed APTT level during day 12 to day 27 (argatroban therapy); (C) AT-III 1 (orange line) showed AT-III level during day 1 to day 3 (UHF therapy); AT-III 2 (blue line) showed AT-III level during day 3 to day 8 ( argatroban therapy); AT-III 3 (green line) showed AT-III level during day 8 to day 12 ( UHF therapy); AT-III 4 (dark blue line) showed AT-III level during day 12 to day 27 (argatroban therapy); (D) platelet 1 (orange line) showed platelet level during day 1 to day 3 (UHF therapy); platelet 2 (blue line) showed platelet level during day 3 to day 8 ( argatroban therapy); platelet 3 (green line) showed platelet level during day 8 to day 12 ( UHF therapy); platelet 4 (dark blue line) showed platelet level during day 12 to day 27 (argatroban therapy).
